# Practical Notes on Diagnosis and Treatment

**Published:** 1909-07-24

**Authors:** 


### PRACTICAL NOTES ON DIAGNOSIS AND TREATMENT.

**The Treatment of Lupus.**

In the treatment of lupus the object to be aimed at is the complete removal or destruction of the diseased tissue. For this purpose internal treatment is useless, although it may sometimes be of service indirectly by remedying any constitutional condition which favours the proliferation of pathogenic micro-organisms.—*Sir Malcolm Morris.*

**Rubber Operating Gloves.**

To my mind, the greatest step in the advance of asepsis in operative surgery is the appreciation of the fact that the skin of the surgeon's hands is one of the chief infecting agencies which patients risk when they submit to a surgical operation.—*Mr. Bland Sutton.*

**The Repair of Fractures.**

It is a mistake to suppose that the exact restitution of the length of the limb is an essential condition for the restoration of muscular power and usefulness. My observations have shown me that some shortening is favourable, rather than the reverse, to muscular efficiency.—*Professor Lucas-Championnière.*

**The Itching of Dry Eczema.**

In later stages, when the eczema has become dry, the following lotion is useful, applied with a brush in a thin coating:—

| | |
|---|---|
| Picis Carbonis ... ... | ʒj. |
| Benzol ... ... ... | ʒiv. |
| Acetone ... ... ... | ℥ij. |

—*Dr. Graham Little.*

